# HOP, a Co-chaperone Involved in Response to Stress in Plants

**DOI:** 10.3389/fpls.2020.591940

**Published:** 2020-10-29

**Authors:** René Toribio, Silvina Mangano, Nuria Fernández-Bautista, Alfonso Muñoz, M. Mar Castellano

**Affiliations:** ^1^Centro de Biotecnología y Genómica de Plantas, Instituto Nacional de Investigación y Tecnología Agraria y Alimentaria (INIA), Universidad Politécnica de Madrid (UPM), Madrid, Spain; ^2^Departamento de Botánica, Ecología y Fisiología Vegetal, Universidad de Córdoba, Córdoba, Spain

**Keywords:** HOP, HSP70, HSP90, co-chaperone, protein folding, quality control

## Abstract

Protein folding is an essential step for protein functionality. In eukaryotes this process is carried out by multiple chaperones that act in a cooperative manner to maintain the proteome homeostasis. Some of these chaperones are assisted during protein folding by different co-chaperones. One of these co-chaperones is HOP, the HSP70-HSP90 organizing protein. This assistant protein, due to its importance, has been deeply analyzed in other eukaryotes, but its function has only recently started to be envisaged in plants. In this kingdom, the role of HOP has been associated to plant response to different cellular, biotic and abiotic stresses. In this article, we analyze the current knowledge about HOP in eukaryotes, paying a special attention to the recently described roles of HOP in plants. In addition, we discuss the recent breakthroughs in the field and the possible new avenues for the study of plant HOP proteins in the future.

## Introduction

### Protein Folding and Chaperones

Protein activity depends on protein conformation and, in this sense, protein folding is an essential step to achieve high activity yields. During translation, amino acids are coupled via peptide bonds to create the polypeptide chain that folds to adopt energetically favorable conformations. Since the nature of amino acid and their arrangement in the protein chain is a major determinant in folding, some polypeptide chains may adopt the protein native conformation by spontaneous folding. However, in many other cases, the acquisition of protein native conformation is assisted by different chaperones that help the formation of the hydrophobic core and the external exposure of the hydrophilic residues (Moran Luengo et al., 2019).

Chaperones belong to five major families that are classified on the basis of their approximate molecular mass and function: small heat shock proteins (sHSPs), HSP60, HSP70, HSP90, and HSP100 (Park and Seo, 2015; Bascos and Landry, 2019). Among them, one of the chaperones most extensively studied in different organisms is HSP70. HSP70 facilitates protein folding by its promiscuous binding to short stretches of hydrophobic residues in the substrate protein, protecting partially unfolded proteins from aggregation and from hydrophobic collapse (Rudiger et al., 1997). In addition, HSP70 actively cooperates with other chaperones in the folding, disaggregation and degradation of substrate proteins (Fernandez-Fernandez and Valpuesta, 2018; Rosenzweig et al., 2019). Specifically, HSP70 and HSP90 act together in the folding of signaling proteins. After HSP70 binding to the most hydrophobic stretches, HSP90 exposes the substrate to a large surface scattered with hydrophobic and charged amino acids, increasing the hydrophobicity and stimulating progression to the native state (Karagoz and Rudiger, 2015; Moran Luengo et al., 2018; Radli and Rudiger, 2018). Despite the fact that, in general terms, HSP90 maintains its activity in the absence of auxiliary proteins (Moran Luengo et al., 2018), the HSP70-HSP90 folding cascade is assisted by different co-chaperones that modulate different aspects of chaperone function as substrate selection, ATPase activity or their capacity to form multiprotein complexes. In such a way, these co-chaperones, in interaction with HSP70, HSP90 or both, facilitate the folding of specific regulatory proteins (Mayer and Bukau, 2005; Li et al., 2012a; Prodromou, 2012; Schopf et al., 2017; Radli and Rudiger, 2018). One of these co-chaperones, which plays an important role in the HSP70-HSP90 folding pathway, is HOP.

### HOP Is a TPR Containing Co-Chaperone That Mediates HSP70/HSP90 Interaction

**FIGURE 1:**
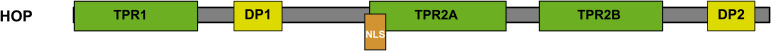
Canonical domain organization of HOP. HOP’s structure usually comprises three TPR domains called (TPR1, TPR2A, and TPR2B), a nuclear localization signal (NLS) that partially overlaps with the TPR2A domain and two DP sequences (DP1 and DP2).

HOP, also known as stress-inducible protein 1 (STI-1), constitutes a conserved family of HSP70/HSP90 co-chaperones in eukaryotes (Nicolet and Craig, 1989; Honore et al., 1992; Blatch et al., 1997; Webb et al., 1997; Demand et al., 1998; Zhang et al., 2003; Song et al., 2009; Hombach et al., 2013). These proteins are characterized by the presence of multiple tetratricopeptide repeat (TPR) motifs, consisting of a loose 34-amino acid consensus sequence, which have been usually involved in protein-protein interactions These TPR motifs are clustered into three TPR domains in the case of HOP (called TPR1, TPR2A, and TPR2B) (Scheufler et al., 2000; Odunuga et al., 2003). In addition, HOP also presents a nuclear localization signal (which partially overlaps with the TPR2A domain), and two conserved sequences containing a tandem of aspartic acid and proline amino acids, namely DP repeats, which are located in the carboxi-terminal region of HOP and seem to affect HOP conformation (Figure 1) (Nelson et al., 2003; Odunuga et al., 2004).

The domains and residues involved in HOP interaction with HSP70 and HSP90 have been well characterized. HOP interaction with HSP70 is supported, on the side of HOP, by the TPR1 domain and, on the side of the chaperone, by the C-terminal HSP70’s sequence GPTIEEVD. Meanwhile, HOP interaction with HSP90 is mediated by HOP’s TPR2A domain and the C-terminal HSP90’s sequence MEEVD (Scheufler et al., 2000; Van Der Spuy et al., 2000; Odunuga et al., 2003). In addition to TPR1 and TPR2A, TPR2B and DP2 domains have been also involved in HOP interaction with the two main chaperones (Odunuga et al., 2004; Song and Masison, 2005; Flom et al., 2007). Finally, the DP2 domain has been speculated to play a role in HSP90 client processing (Schmid et al., 2012).

**FIGURE 2:**
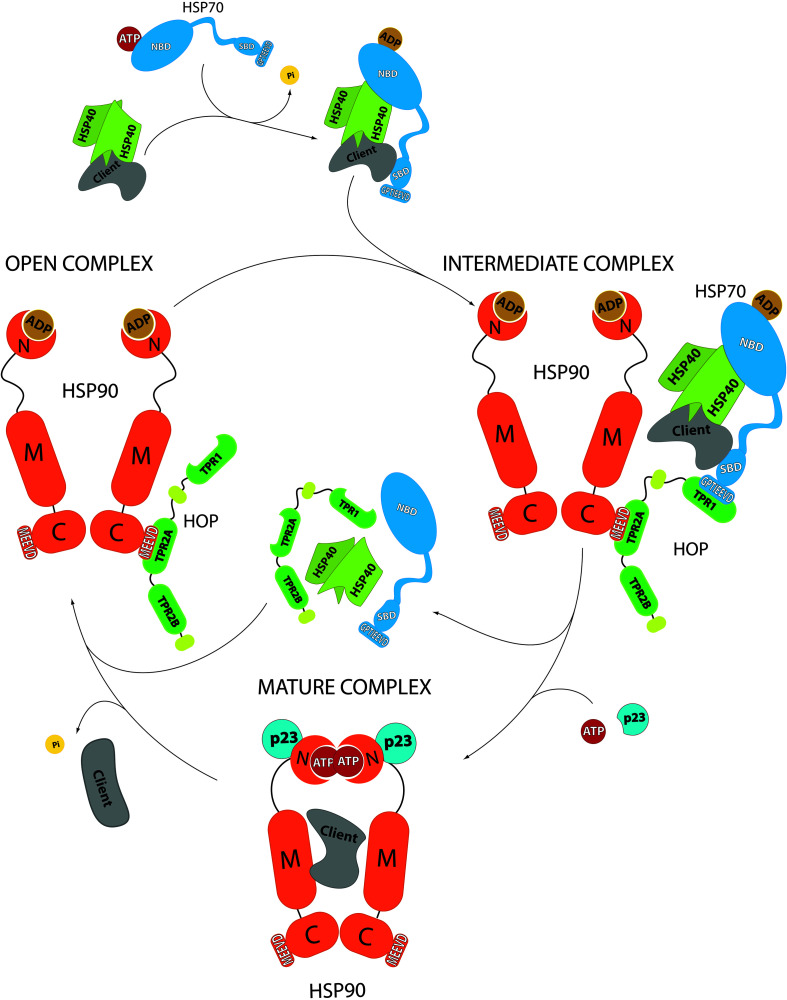
Role of HOP in the protein folding cycle by HSP70-HSP90 according to GR folding. In an early stage, the client protein associates to HSP40 and HSP70, leading to HSP70’s ATP hydrolysis. In this complex, HSP70-ADP conformation favors the association with HOP (Alvira et al., 2014) (which is already in association with HSP90). In this complex, called intermediate complex, HOP serves as a “bridge” that brings in close proximity HSP70 and HSP90, favoring the transfer of the client protein from HSP70 to HSP90. ATP binding to HSP90 prompts a conformational change that leads to the release of HOP, HSP70 and its co-chaperones and the generation of the mature complex. This last complex, where the co-chaperone p23 gets involved, allows the final maturation of the client protein, which is released after ATP hydrolysis and HSP90’s return to its open state. NBD: HSP70’s N-terminal ATPase domain. SDB: HSP70’s substrate binding domain. GPTIEEVD: HSP70’s sequence for binding to HOP. N: HSP90’s N-terminal domain. M: HSP90’s middle domain. C: HSP90’s C-terminal domain. MEEVD: HSP90’s sequence for interaction with HOP.

The molecular function of HOP within the HSP70-HSP90 folding pathway involves the generation of a molecular bridge that facilitates the transfer of the substrate protein from HSP70 to HSP90 through the simultaneous binding to both chaperones. The role of HOP in this pathway has been deeply studied in the context of the folding of the glucocorticoid receptor (GR), where HOP is not strictly required for GR folding, but significantly increases the yield in the acquisition of GR’s native conformation (Morishima et al., 2000). GR’s activity is regulated through its ligand binding domain (LBD), since binding of the ligand promotes structural stability and the allosteric control that allows the interaction of the GR with co-regulator proteins to modulate transcription (Bain et al., 2007). In this context, HOP interacts simultaneously with pre-existing HSP70-GR complexes and with HSP90, in such a way that GR’s LBD is delivered in the proximity to HSP90’s client binding site to allow ligand binding and function. This pathway, in which HOP plays a main role, is also assisted by other co-chaperones that bind to HSP70 or to HSP90 (Figure 2). In addition to its function as a molecular bridge, HOP seems to indirectly regulate the binding of other co-chaperones to the HSP70-HSP90 complex, as it is the case for FKBPs, CYP40 or AHA1, whose interactions with HSP90 are inhibited in the presence of HOP (Owens-Grillo et al., 1996; Harst et al., 2005; Ebong et al., 2016).

Finally, it is worth to note that HOP interaction with other chaperones have been also reported, suggesting that HOP may assist other chaperone complexes that may be involved in HSP70- and HSP90-independent processes (Gebauer et al., 1998; Abbas-Terki et al., 2001; Zanata et al., 2002).

### Role of HOP in Non-Plant Eukaryotes

HOP, also initially named STI1 for stress-inducible protein 1, was firstly identified as a heat stress transacting factor in yeast (Nicolet and Craig, 1989). In these eukaryotes, cells carrying a disruption in *HOP/STI1* showed an increase in doubling time at 37 °C, suggesting a role in heat response. In addition, a possible role in thermotolerance was also described in *Caenorhabditis elegans.* CsHOP lacks the TPR1 domain; nevertheless, a knock-out mutation in this non-canonical isoform of HOP leaded to a reduction in brood size and worms’ survival under extreme heat conditions (Song et al., 2009).

In mice, HOP/STI1 is strictly required during embryo development, meanwhile different attempts to reduce the expression of *HOP/STI1* in adult mice revealed multiple phenotypes, including increased sensitivity to cerebral ischemia, reduced hippocampal neuronal resilience during aging, reduced attention and increased hyperactivity (Beraldo et al., 2015; Lackie et al., 2017).

In addition, recent studies have demonstrated that STI1/HOP levels are increased in a large number of cancer cell types (Sun et al., 2007; Walsh et al., 2009, 2011; Kubota et al., 2010; Wang et al., 2010; Sims et al., 2011; Tsai et al., 2012), and that the increased STI1/HOP function is associated with cancer cell proliferation and migration (Kubota et al., 2010; Horibe et al., 2011, 2012; Li et al., 2012b; Willmer et al., 2013). Moreover, HOP/STI1 has been involved in neuritogenesis and in neuroprotection against degenerative diseases as Alzheimer’s disease and prionopathies (Bohush et al., 2019).

Despite the fact that the precise involvement of HOP in each of those specific processes is not known, it is highly probable that it could be related to its role in protein folding. It is worth to note that heat courses with protein denaturation. In addition, unfolded proteins form aggregates, which are characteristic of different cancers and neurodegenerative diseases (Bohush et al., 2019). Furthermore, STI1/HOP has been shown to interact and form complexes with HSP90 in some cancer cells, suggesting that HOP function in cancer could be associated to alterations in the function of the HSP70/HSP90 complex (Kubota et al., 2010). This conclusion is supported by studies that show that the disturbance in HOP’s interaction within the HSP70-HOP-HSP90 complex reduces cancer cell proliferation (Horibe et al., 2011, 2012).

### HOP Function in Plants Is Currently Mainly Associated With the Plant Response to Stress Conditions

HOP homologs have been described in different plants (Zhang et al., 2003; Nakashima et al., 2008; Chen et al., 2010; Prasad et al., 2010; Fellerer et al., 2011; Xu et al., 2014). In some species, HOP is encoded by multi-gene families. In this sense, analysis of different databases retrieves only one HOP member in barley and tomato; two members are identified in rapeseed and soybean, three members are present in Arabidopsis and maize, and six members have been recently identified in *Triticum aestivum* (Table 1 and Meena et al., 2020).

**TABLE 1:** HOP homologs currently identified in the database in different plant species.

**Organism**	**Members**	**Protein ID**
*Arabidopsis thaliana*	3	Q9LNB6.1 (AtHOPI), Q5XEP2.1 (AtHOP2), Q9STH1.1 (AtHOP3)
*Brassica napus*	2	XP_013698107.1, XP_013695244.1
*Brassica rapa*	2	XP_009113054.1, XP_009144494.1
*Camelina sativa*	3	XP_010476198.1, XP_019093490.1, XP_010455678.1
*Citrus clementina*	1	XP_006440382.1
*Citrus sinensis*	1	XP_006477255.1
*Coffea arabica*	1	XP_027079914.1
*Elaeis guineensis*	1	XP_010933724.1
*Glycine max*	2	NP_001236261.2, XP_003538668.1
*Gossypium hirsutum*	1	XP_016709846.1
*Helianthus annuus*	2	XP_022002222.1, XP_022029610.1
*Hordeum vulgare*	1	KAE8796582.1
*Malus domestica*	1	XP_008349064.2
*Manihot esculenta*	1	XP_021604177.1
*Nicotiana attenuata*	2	OIT04983.1, OIT38257.1,
*Nicotiana tabacum*	2	XP_016505277.1, XP_016460758.1
*Olea europaea*	1	XP_022881121.1
*Physcomitrella patens*	1	XP_024380708.1
*Pistacia vera*	1	XP_031247860.1
*Prunus avium*	1	XP_021810314.1
*Prunus mume*	1	XP_008238627.1
*Prunus persica*	1	XP_007210502.1
*Raphanus sativus*	2	XP_018458658.1, XP_018456252.1
*Solanum lycopersicum*	1	XP_004245731.1,
*Solanum tuberosum*	2	XP_006358357.1, XP_006355497.1
*Vitis vinifera*	1	RVX15633.1
*Zea mays*	3	PWZ38312.1, PWZ26136.1, PWZ20685.1

*All the results were curated using The Basic Local Alignment Search Tool (BLAST) against the AtHOP to ensure high homology to the HOP family.*

In Arabidopsis, the three members of the family (AtHOP1, AtHOP2, and AtHOP3) share high homology and a similar domain structure to its human and yeast counterparts (Figure 1), and indeed, consistent with the high conservation of the TPR2A domains, these three proteins were shown to interact *in vivo* with HSP90 (Fernandez-Bautista et al., 2017a, 2018). These HOP proteins also contain a conserved bipartite nuclear localization signal. In this regard, it has been demonstrated that AtHOP1, AtHOP2, and AtHOP3 are mainly localized in the cytoplasm under control conditions, but that all these proteins partially accumulate in the nucleus and stress granules (SGs) under heat stress conditions (Fernandez-Bautista et al., 2018). However, despite these similarities, *HOP* genes display a differential pattern of expression in Arabidopsis; while *AtHOP1* and At*HOP2* seem to be constitutively expressed in different tissues (with either no or modest induction under the tested environmental challenges), the expression of *AtHOP3* is modestly induced under endoplasmic reticulum (ER) stress conditions and highly induced under heat challenges (Fernandez-Bautista et al., 2017a, 2018).

In addition to its main localization in the cytoplasm, AtHOP3 also co-localizes with ER marker proteins under control conditions, suggesting that HOP3 is also found and can play a role in the ER (Fernandez-Bautista et al., 2017a). This observation was further supported by the *in vivo* interaction of HOP3 with BiP, a major HSP70 chaperone with a strict ER localization. BiP plays a main role in the folding of proteins in the ER and in the alleviation of the ER stress response associated to the accumulation of misfolded proteins in this compartment. Consistent with the interaction with BiP and BiP’s role, *hop3* loss-of-function mutants show a hypersensitive phenotype in the presence of ER stress inducers such as dithiothreitol (DTT) and tunicamycin (TM), a phenotype that is reverted to the wild-type situation by the addition of the chemical co-chaperone tauroursodeoxycholic acid (TUDCA). Based on these data, it has been speculated that HOP3 plays a major role along with BiP in the folding of proteins in the ER, alleviating the ER stress response. This effect seems independent of the proper establishment of the unfolded protein response (UPR), since *hop3* mutants do not show a clear alteration in the transcription of UPR marker genes (Fernandez-Bautista et al., 2017a).

Regarding the role of AtHOP3 in the ER stress response, it has to be considered that multitude of processes, such as specific developmental programs and the efficient response to different stress conditions, require the synthesis and folding of a large number of proteins that should be transported to the plasma membrane or secreted. During these processes, the demand for folding of these membrane and secreted proteins overcomes the basal folding capacity of the ER, prompting the so-called ER stress (Deng et al., 2013; Liu and Howell, 2016). In this regard, the involvement of AtHOP3 in the alleviation of the ER opens the possibility that HOP3 may also participate in processes that course with an intrinsic ER stress (Fernandez-Bautista et al., 2017b). As it seems to be the case of pollen germination in Arabidopsis (Fernandez-Bautista et al., 2017a).

Moreover, the role of HOP has been also analyzed during the response to different pathogens such as viruses and fungi (Chen et al., 2010; Xu et al., 2014; Lamm et al., 2017). During some pathogen-plant interactions, pattern recognition receptors (PRRs) sense the conserved microbe associated molecular patterns (MAMPs), initiating the defense response (Boller and Felix, 2009; Noman et al., 2019). One example of a well-known PRR is the CHITIN ELICITOR RECEPTOR KINASE 1 (CERK1), which recognizes the MAMP chitin from the fungal cell wall (Miya et al., 2007). CERK1 belongs to the family of lysine-motif receptor-like kinases (RLKs) and it is folded in the ER and transported to the plasma membrane. In this sense, and in accordance with AtHOP3 localization in the ER, it has been described that OsHOP/STI1a interacts with HSP90 and OsCERK1 in the ER (Chen et al., 2010). Through this interaction, HOP has been involved in the ER maturation of CERK1 and in regulating the transport from the ER to the plasma membrane through a SAR1-dependent vesicle trafficking pathway. Once on the membrane, rice HOP/STI1a forms part of large protein complexes along with CERK1, RAC1 and HSP90 called the defensome. The fundamental role of HOP/STI1a in plant defense against this fungus in rice was further highlighted by the increased susceptibility of HOP/STI1 RNAi lines and the higher resistance of the HOP/STI1 overexpressing plants to rice blast infection (Chen et al., 2010).

Interestingly, not every single PRR seems to strictly require HOP for its folding and maturation. Indeed, through the generation of HOP/STI1 RNAi lines in *Nicotiana tabacum*, Lamm and coworkers demonstrated that, despite the fact that these plants were unable to respond to chitin treatment (probably due to defects in CERK1 maturation), the trafficking and functionality of FLAGELLIN SENSING 2 (FLS2, a different well-known PRR) were unaffected, suggesting that HOP could somehow display specificity for certain substrates (Lamm et al., 2017). Using these lines, this group also provided additional information of the role of HOP/STI1 during Potato virus Y (PVY) viral infection. In general terms, the interaction between PVY^*N*^ and *N. tabacum* cv. Samsun NN leads to veinal necrosis in stem and leaves. However, these symptoms were not observed in the HOP/STI1 RNAi lines, even though PVY was able to accumulate to almost wild-type levels during the infection. This lack of symptomatology was also accompanied with a reduction in the transcriptional induction of pathogenesis-related *(PR)* genes and in the salicylic acid (SA) accumulation, which further indicated an altered establishment of the defense response. Based on these data, HOP/STI1 was proposed to participate in the virus perception and the activation of plant viral defense (Lamm et al., 2017). During the infection, HOP was localized in ER-derived viral aggregates that are compatible with viral replication complexes, but, in accordance with previously reported data in Arabidopsis (Fernandez-Bautista et al., 2017a), the RNAi lines did not seem to display a clear alteration in the UPR (Lamm et al., 2017). Altogether, the data suggest that HOP is involved in the folding of specific proteins in the ER and modulates viral defense, but does not seem to modify specifically the UPR response. In addition, its possible localization in viral replication complexes during PVY infection in tobacco plants may also suggest that HOP could be somehow involved in viral replication or translation (Lamm et al., 2017). This aspect, although it was not further analyzed in the former virus-plant system, was explored during the mitochondrial Carnation Italian ringspot tombusvirus (CIRV) interaction with *N. benthamiana* (Xu et al., 2014). In this plant-pathogen system HOP interacts with the viral protein p36 and seems to act as a restriction element for virus replication. Interestingly, this effect seems quite specific, since peroxisome membrane-based replication of the closely related Tomato bushy stunt virus (TBSV) or Cucumber necrosis virus (CNV) are not affected by the presence of HOP (Xu et al., 2014).

Furthermore, the role of HOP family in acquisition of long-term acquired thermotolerance (LAT) was also analyzed in Arabidopsis (Fernandez-Bautista et al., 2018). Surprisingly, despite *HOP3* being the only member of the family highly induced under high temperature conditions, which suggested a relevant role of this specific protein under heat stress, the tolerance analyses in the *hop* single- and triple-mutant/s point out a partial redundant role of the three members of HOP in Arabidopsis in LAT establishment. The role of HOP in this process seems to be dual. In the one hand, during heat stress HOP proteins shuttle from the cytoplasm to the nucleus and seems to modulate the proper activation of the transcriptional heat stress response (HSR). This hypothesis is based on the subcellular localization of the HOP members and on the altered transcription of more than a hundred heat-responsive genes in the mutant plants during the acclimation period. On the other hand, an unusual high accumulation of insoluble and ubiquitinated proteins is observed in the *hop1 hop2 hop3* triple mutant at high temperature, which suggests that HOPs play a main role in the quality control (QC) of the cytoplasmic proteins under heat (Fernandez-Bautista et al., 2018). In adition, quite recently, a functional role of TaSTI-2A in thermotolerance acquisition has been also reported in wheat (Meena et al., 2020).

Finally, the association of HOP along with HSP90 with pre-proteins synthesized in wheat germ extract suggests that HOP has a role in the maintaining of these proteins in a competent state until the moment they are imported into the chloroplast (Fellerer et al., 2011).

**FIGURE 3:**
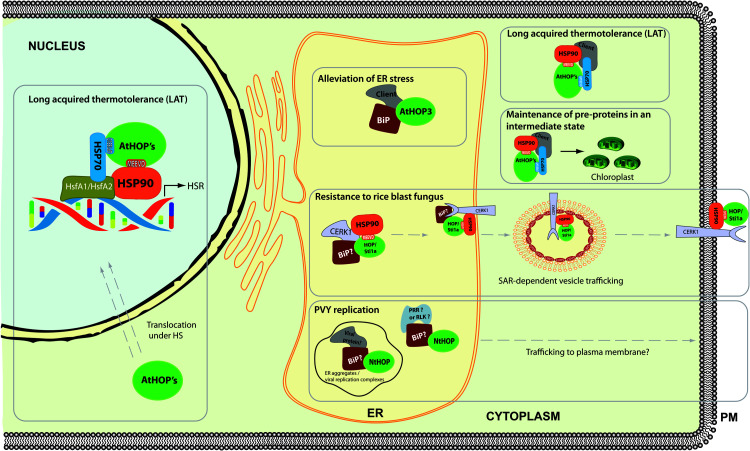
Roles of HOP in plants. This model compiles the known information of the role of HOP in plants, highlighting the role of this co-chaperone in plant response to cellular and environmental stresses.

A summary of all the reported functions of HOP in plants to date can be found in Figure 3.

## Future Challenges and Perspectives

HOP is a central co-chaperone within the HSP70-HSP90 cycle and, in this sense, it seems quite possible that HOP could be involved in the folding and in the stability of multiple proteins. Based on this, we speculate that HOP proteins could be implicated in multiple processes. In this regard, understanding the full function of HOP in plants will require the identification of the substrate proteins that require HOP for their efficient folding and a close inspection of the phenotypes of the *hop* mutants. As described before, in many plant species, HOP proteins are encoded by multiple genes. Moreover, in some cases, as it is the case of Arabidopsis, different members of the family seem to have different tissue expression and only partial redundancy. This effect is highlighted in the case of *hop3* mutants, which show a clear phenotype under ER stress conditions in Arabidopsis with fully functional HOP1 and HOP2. This gene complexity will entail, depending on the species, the deep analysis of the different *HOP* genes and a careful phenotypical analysis of single and multiple mutants.

The studies carried out in *Nicotiana* and rice suggest that HOP modulates the folding of CERK1, but not of FSL2 (Chen et al., 2010; Lamm et al., 2017). Given that both molecules constitute PRRs involved in plant defense, these analyses suggest that HOP could have certain substrate specificity. In this regard, another aspect that could highly contribute to understand the role of HOP, not only in plants but also in other eukaryotes, will be the analysis of HOP specificity within the targets of HSP90. As described before, not all proteins need the assistance of co-chaperones for folding, which suggests that not all HSP90 targets require HOP. In addition, it has been described that the binding of HOP excludes the binding of other co-chaperones to HSP90 in other organisms, which allows to speculate that different co-chaperones may assist the folding of different proteins or protein structures.

Moreover, it is possible that this specificity may vary depending on the developmental and stress conditions. Under some conditions, which do not course with a high pressure on folding capacity, HOP and other co-chaperones may be strictly needed for the folding of specific proteins. However, it could be also envisaged that under certain cellular or environmental stresses, such as under ER- or heat-stress conditions, which highly exceed the folding capacity of the cells, HOP may assist the folding of a larger number of proteins. This may have helped to easily identify the role of HOP in response to stress, but, based on the information in other eukaryotes, it is highly possible that HOP could modulate the folding of specific proteins, such as kinases and specific receptors. Therefore, the analysis of the role of HOP during development (in the absence of harsh challenges) constitutes an interesting aspect that remains unstudied in plants.

From the mechanistic point of view, the knowledge of the role of HOP in the interaction with HSP70, HSP90 and during the transfer of proteins from one to the other is extremely scarce in plants compared to other organisms. This study would help to understand HOP function in plants and to establish possible similarities and differences with HOP proteins in other organisms.

Finally, another intriguing question is the exact role of HOP in the folding of the different targets. In general terms, proteins start their folding either in the ER or in the cytoplasm, locations where HOP is localized. However, HOPs have also been observed in other locations, such as in the nucleus, trafficking through the Golgi and in extracellular complexes. In these locations, HOP role in folding assistance may include the maintenance of proteins in an intermediate folding state while they reach their final destination (as it has been observed for proteins transported to the chloroplast) or while they bind to other co-factors to achieve their functional conformation. In this regard, identification of substrate-HOP complexes, along with the study of their localization, may allow us to increase our knowledge of the precise mechanism of HOP in protein folding and maturation.

## Author Contributions

All authors listed have made a substantial, direct and intellectual contribution to the work, and approved it for publication.

## Conflict of Interest

The authors declare that the research was conducted in the absence of any commercial or financial relationships that could be construed as a potential conflict of interest.
